# Applying a Social Ecological Model to Medical Legal Partnerships Practice and Research

**DOI:** 10.1017/jme.2023.151

**Published:** 2023

**Authors:** Susan McLaren, Lisa Radtke Bliss, Christina Scott, Pam Kraidler, Robert Pettignano

**Affiliations:** 1: GEORGIA HEALTH POLICY CENTER, ANDREW YOUNG SCHOOL OF POLICY STUDIES AT GEORGIA STATE UNIVERSITY, ATLANTA, GA, USA; 2: GEORGIA STATE UNIVERSITY COLLEGE OF LAW, ATLANTA, GA, USA; 3: ATLANTA LEGAL AID SOCIETY, ATLANTA, GA, USA; 4: EMORY UNIVERSITY SCHOOL OF MEDICINE, ATLANTA, GA, USA.

**Keywords:** Medical Legal Partnership, Social Determinants Of Health, Social Ecological Model, Evaluation, Outcomes, Health Harming Legal Needs

## Abstract

The social ecological model (SEM) is a conceptual framework that recognizes individuals function within multiple interactive systems and contextual environments that influence their health. Medical Legal Partnerships (MLPs) address the social determinants of health through partnerships between health providers and civil legal services. This paper explores how the conceptual framework of SEM can be applied to the MLP model, which also uses a multidimensional approach to address an individual’s social determinants of health.

Medical legal partnerships (MLPs) bring together professional expertise in law, health, and other disciplines to address patients’ health harming^1^ legal needs (HHLN). MLPs address social determinants of health (SDOH) through partnerships between health care providers and civil legal service providers.^2^ SDOH are the “conditions in which people are born, grow, work, live, and age, and the wider set of forces and systems shaping the conditions of daily life.”^3^ SDOH adversely impact health outcomes, and the U.S. ranks as one of the worst among economically advanced nations in addressing health outcomes.^4^ This low ranking reflects limited investments in social, economic, and environmental factors that contribute to health disparities and poor health outcomes.^5^ MLPs use multiple practices and intervention strategies including direct legal services, education, research, and systemic advocacy to address factors contributing to poor health.^6^

This article proposes that MLPs use the SEM to guide program design, evaluation, and research. The article begins with an overview of SDOH and SEM. Next, it explores how the SEM may be applied to illustrate how MLP interventions improve individual health by providing direct legal services to address individual patient needs. Additionally, it explores how MLPs support transformational system changes at the organization, community, and policy levels through interdisciplinary education, interprofessional collaboration, research, and systemic advocacy.

The social ecological model (SEM) is a conceptual framework that can be applied to MLP practice and research to help MLP collaborators better understand the impact level of their activities and the ways in which their interventions address patient/client outcomes. The SEM framework recognizes that individuals function within multiple interactive systems and contextual environments that influence their health. SEM can guide communities in the design of public health interventions to address health concerns^7^ generally and health improvement programs that address SDOH specifically, such as MLPs.^8^ While typical MLP intervention strategies align with the contextual environmental levels identified by SEM, little literature exists that applies SEM to MLPs (also known as “Health Justice Partnerships” in other countries), and none is known to be applied to U.S.-based MLP practice or research findings. Thus, MLPs are ripe for examination using the SEM approach.^9^

This article proposes that MLPs use the SEM to guide program design, evaluation, and research. The article begins with an overview of SDOH and SEM. Next, it explores how the SEM may be applied to illustrate how MLP interventions improve individual health by providing direct legal services to address individual patient needs. Additionally, it explores how MLPs support transformational system changes at the organization, community, and policy levels through interdisciplinary education, interprofessional collaboration, research, and systemic advocacy. The authors illustrate the application of the SEM using examples from the activities of the Health Law Partnership (“HeLP”), an academic medical legal partnership based in Atlanta, Georgia. This exercise demonstrates that MLP components collectively influence SDOH through an interconnected systems approach. Finally, applying the SEM to MLPs shows its use as a tool for MLPs to target their activities to maximize impact on health outcomes across systems and environments.

## Social Determinants of Health and the Social Ecological Model

Over the last two decades, public health professionals have realized that health is influenced by broad, interrelated components beyond medical care, behavior, and genetics; it is also impacted by environmental and physical influences and SDOH.^10^ In the U.S., health disparities persist in health care coverage, access to health care, morbidity, and mortality for minority and vulnerable populations.^11^ Addressing SDOH is critical to improving health and reducing health disparities.^12^ SDOH are influenced by social, economic, environmental, and structural factors including governing systems and policies that affect the equitable distribution of resources in society that result in health inequities and lead to health disparities among different communities and populations.^13^ The U.S. Office of Disease Prevention and Health Promotion has identified five domains for examining SDOH: economic stability, education access and quality, health care access and quality, social and community context, and neighborhood and built environments.^14^

While examining SDOH highlights the social, economic, environmental, and structural factors contributing to health disparities, the SEM provides a roadmap for understanding how multiple systems may interact to influence health outcomes. Urie Bronfenbrenner expanded the ecological systems theory from its early development by Kurt Lewin in the mid-20th century,^15^ theorizing that individuals grow and develop within an ecological system that contains multiple environments that interact to shape them. The theory illustrated this interdependent influence using overlapping concentric circles with the individual at the center. The closer the layers are to the individual, the more influence that system has on their experiences.^16^ K.R. McLeroy and others redefined SEM as a framework to promote health-related behavioral change that places the individual or target population at the center.^17^

As a conceptual framework, SEM can be used to delineate the interrelated components of individual and contextual environments that influence health across five core constructs that impact SDOH: (1) intrapersonal, (2) interpersonal, (3) institution/organization, (4) community and (5) societal.^18^ The intrapersonal level reflects an individual’s knowledge, attitudes, beliefs, and skills that influence health. The interpersonal level of SEM reflects the individual’s relationships with family, friends, and social networks. At the institutional/organization level, mechanisms that influence health are the environments in which individuals interact and live their lives, such as neighborhoods, work, schools, and other institutions with which individuals engage. At the community level, SEM considers the networks and relationships among institutions and organizations which influence the settings where individuals live and interact. Community structures influence how people behave based on resources available in the built environment and cultural norms.^19^ Finally, an individual’s health is influenced at the policy and societal level by governmental policies, laws and regulations that result in broader system changes affecting health and wellbeing.^20^ Because the health interventions or changes made at the intra-/interpersonal and organization/institution levels directly involve the individual, they often result in first-order changes that address individual health needs. Interventions at the community level and above often spawn more transformative systemic change that benefits both an individual’s and a population’s health because they are structural interventions that more broadly address SDOH (i.e., governing systems and policies that affect equitable resource distribution).^21^

While the SEM illustrates the broad factors or constructs that influence health, its central tenet is to encourage the combination of interventions at the individual (intrapersonal/interpersonal), environmental (organizational/community), and policy (societal) levels in order to lead to sustainable positive changes in health.^22^ Depending on the issue being addressed, researchers have adapted the model to include additional constructs (i.e. sub-individual levels like genetics, or further delineation of environmental factors) or collapsed constructs for broader applicability across different health promotion and prevention interventions.^23^ The CDC uses a four-construct SEM that recognizes the individual, relationships, community, and society as a framework for mapping prevention strategies and interventions.^24^ The SEM also provides a framework for integrating multiple theories and models to support a comprehensive approach to designing, implementing, and evaluating health-improving policies and interventions by addressing barriers to health. Consequently, the SEM has been increasingly used by the World Health Organization and the U.S. Department of Health and Human Services when developing health improvement interventions. Empirical research and systematic reviews of the SEM framework show its use in interventions in health literacy, health equity, health disparities training for medical students, physical activity, and domestic violence prevention.^25^

## Applying the SEM to MLPs

**Figure 1.**
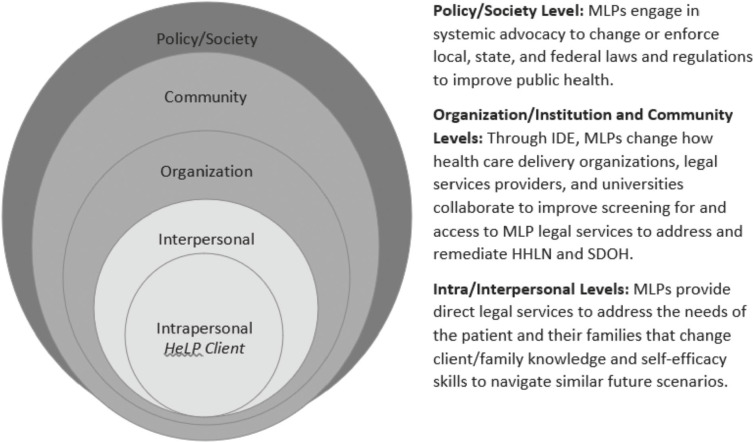
Social-ecological model for illustrating how medical legal partnerships (MLPs) address health harming legal needs (HHLN) and social determinants of health (SDOH) that adversely impact health.

MLPs are public health interventions that use a multi-dimensional approach to address SDOH that can be understood by applying the SEM. However, systematic reviews of published literature underscore the need for more theory-driven and rigorous research studies to establish the evidence base for the impact of MLPs on SDOH and health outcomes across all aspects of the intervention^26^ to justify expansion of financing structures for MLP program sustainability. In Figure 1, the SEM is adapted to illustrate and summarize how MLPs address HHLN and SDOH that adversely impact health. The SEM is adapted to MLPs using the five core constructs of the model, combining the intra and interpersonal levels, and the organizational and community levels. As shown in Figure 1., MLP components such as providing direct legal services to clients, interdisciplinary education (IDE) and interprofessional collaboration (IPC) among medical and legal professionals, and systemic advocacy in the form of addressing laws that affect SDOH can influence causal pathways that impact the health of MLP clients, their families, and communities.^27^

To illustrate the application of the SEM to MLP, the authors use the Health Law Partnership (HeLP), an academic MLP based in Atlanta, Georgia, whose activities include public health legal services delivery, interprofessional education, advocacy, and evaluation and research.^28^ HeLP is a collaboration among Georgia State University College of Law, Atlanta Legal Aid Society, and Children’s Healthcare of Atlanta. At the intrapersonal and interpersonal levels of the SEM, MLPs like HeLP provide free civil legal services and educate clients and families on how to recognize and navigate HHLN by helping them understand their rights and the structural processes for addressing the health impeding legal issues. HeLP’s public health legal services range from brief self-help advice to clients to legal representation in a variety of civil legal matters that have a connection to health and well-being.

HeLP’s engagement at the intra-/interpersonal levels has resulted in documented positive changes to clients’ knowledge, attitudes, and skills that equip and empower them to successfully advocate for themselves and their families, navigate systems, and handle similar future problems as reported through HeLP client pre/post-intervention surveys.^29^ Additionally, HeLP clients have rated how they view their own (intrapersonal) and their family’s (interpersonal) physical and emotional health, physical safety, and financial and family well-being. Pre/post-intervention surveys compare clients’ perceptions of changes in knowledge and skills that support intrapersonal needs such as the way clients understand legal rights and what important documents to retain; believe that they can discuss important health issues with medical professionals; access health services and social benefits; and deal with stress. Post-surveys ask clients about intrapersonal outcomes such as whether HeLP improved their ability to handle similar future problems. Across the board, clients report improvement in almost every intra- and interpersonal category following MLP intervention.^30^ Additionally, HeLP and other MLPs’ legal services have resulted in direct benefits to clients and their families that may include compensation, access to services and resources (i.e. benefits such as SSI, Medicaid, housing, education services) or remediation from unsafe environments. These improve client and family health and health outcomes.^31^ Additionally, observational studies of MLPs, mostly using pre-post design, have also reported improvements in client-reported health status, stress, self-efficacy, and satisfaction with outcomes as a result of receiving MLP services.^32^ These findings suggest that access to HeLP, and MLPs generally, positively affect clients and families, including their ability to manage health and HHLN, at the intra- and interpersonal levels of the SEM. Nevertheless, such success may be difficult to quantify.

At the institutional/organization and community levels of SEM, MLPs influence the settings in which clients and their families live and receive care and services (i.e., health care clinics, hospitals, schools, neighborhoods). By incorporating MLP services into the blueprint of health care delivery, organizations with MLPs are adopting interprofessional collaboration (IPC) strategies and policies to address HHLN/ SDOH that adversely impact client health and health outcomes. These strategies improve the settings in which clients and families receive services. IPC relies upon professionals from the legal and health care delivery disciplines implementing interdisciplinary education (IDE) strategies and organizational policies and procedures that promote knowledge and awareness among providers of the HHLN/SDOH and increasing recognition of and screening of clients for HHLN/SDOH.

MLPs positively influence the systems in which they operate, thereby benefiting the health and wellbeing of individuals who interact with them. For example, since MLPs were established in the early 1990s, observational research has found that MLPs increase screenings for SDOH as HHLN that can adversely exacerbate health issues^33^, increase access to free MLP civil legal services^34^; and address health harming legal needs related to health care, government/social benefits, housing/utilities, income security, education, and employment services.^35^

Through professional student IDE, academic MLPs also have the potential to prospectively influence the environments in which individuals receive care and services that influence health and health outcomes. For example, HeLP’s law and medical student surveys indicate that HeLP has improved their interprofessional communication, collaboration, and ability to meet patient’s/client’s needs.^36^ MLPs that include law school and/or medical school partners implementing formal IDE and experiential training opportunities for residents and professional students (i.e., law, medicine, social work, nursing, public health) promote IPC and problem-solving to remediate HHLN and SDOH. HeLP’s interdisciplinary education, as measured through pre- and post-surveys, has resulted in positive changes in professional student knowledge, skills, and attitudes about screening for and communication and collaborating with other disciplines to address HHLN/SDOH^37^ and meet patients and clients’ needs.^38^ Among fourth-year medical students who participate in HeLP IDE, post-survey results indicate that 50 percent more students believed that it is very important or important to refer a patient who is experiencing socioeconomic barriers to a MLP legal professional than they did when given the pre-survey.^38^ This suggests that MLP activities at the institutional/organization and community levels influence the contexts in which individuals engage with systems when MLP is part of the delivery model.

Finally, at the SEM policy/societal level, MLPs engage in systemic advocacy to change or enforce governmental laws and regulations to improve public health. Since its inception, HeLP has prioritized systemic advocacy. Some examples of issues it advocated for are policy changes raising the age for booster seat and required flotation device use, decreasing the blood alcohol content limit for boating, improving the Medicaid Administrative Appeals processes, and improving local housing conditions.^40^ Other MLPs have advocated to change federal subsidized housing laws and regulations that protect low-income families from lead exposure, protect insurance coverage from being eliminated under the federal exchange for low-income residents in the Washington D.C. area, and to correct procedures that violated federal regulations and hindered access to medical and food stamp benefits.^41^

As a result of the growing research that continues to demonstrate the impact of MLPs on HHLN/SDOH, federal agencies including the Centers for Disease Control and Prevention and Human Resources and Services Administration (HRSA) have modified policies to support their adoption. HRSA has modified funding policies to support reimbursement for MLP legal services as enabling services to improving health.^42^ Similarly, the value of MLP IDE and IPC has been recognized by the Association of American Medical Colleges, which in 2015 funded the development of the “Accelerating Health Equity, Advancing through Discovery Medical-Legal Partnership Learner Pre/Post Survey” to support the evaluation of four “entrustable professional activities” and six “general physician competencies” across different educational intervention models as an upstream strategy for addressing SDOH and improving health equity and outcomes.^43^ These policy/society level activities contribute to public health more broadly, affecting not only the individual, but society at large.

Health outcomes evaluation using the SEM can be complex because of the multi-dimensional components that influence SDOH. This includes the ability to link health and social services and policy data across interventional components to individual health. Consequently, it is difficult to demonstrate the impact of MLP on SDOH and health outcomes. Most published MLP studies are reliant on observational designs that utilize MLP participant-reported outcomes through surveys or qualitative interviews, legal outcomes data, or administrative claims or electronic health data available through the medical partner. Limited access to health data and public data sets can inhibit programs from assessing the broader systemic impacts of MLPs at the societal levels. Limits on data and funding challenge MLPs to apply rigorous evaluations that include a comparison group to demonstrate MLP impacts. In the absence of research evidence to demonstrate the effect of MLPs across the SEM, systematic funding for the intervention remains deficient.

## Conclusion

The body of research on the impact MLPs have on SDOH is growing; though research documenting the extent to which MLPs address health disparities across SEM is limited. Thus far, research has focused on describing short-term outcomes like legal needs screenings, access to legal services, resolution of individual-level SDOH health harming issues, individual health outcomes, and health system cost savings and return on investments. Applying SEM categories to MLPs can illuminate potential gaps in interventions as well as identify areas to measure program outcomes. Using the SEM lens, MLPs can examine the scope of their interventions within each of the SEM levels. If an MLP is not targeting each level of SEM, it can design supplemental activities or interventions in other levels to maximize opportunities for improved health outcomes. SEM is a mechanism for articulating interventional elements of MLP IPC designed to address SDOH across the individual, organizational, community, and policy levels. Additionally, SEM provides a framework for MLPs to disseminate findings to broader audiences which can support long term model sustainability and scalability. Through SEM, MLPs can ensure their strategies for addressing and improving SDOH and health outcomes are comprehensive and cross different dimensions. This understanding can help all MLPs assess and reimagine their potential for impact in multiple spheres.

## Note

The authors have no conflicts to disclose.
